# Deletion of *Prepl* Causes Growth Impairment and Hypotonia in Mice

**DOI:** 10.1371/journal.pone.0089160

**Published:** 2014-02-28

**Authors:** Anna Mari Lone, Mathias Leidl, Amanda K. McFedries, James W. Horner, John Creemers, Alan Saghatelian

**Affiliations:** 1 Department of Chemistry and Chemical Biology, Harvard University, Cambridge, Massachusetts, United States of America; 2 Belfer Institute for Applied Cancer Science, Dana-Farber Cancer Institute, Harvard Medical School, Boston, Massachusetts, United States of America; 3 Department of Medical Oncology, Dana-Farber Cancer Institute, Harvard Medical School, Boston, Massachusetts, United States of America; 4 Institute for Applied Cancer Science, University of Texas MD Anderson Cancer Center, Houston, Texas, United States of America; 5 Laboratory for Biochemical Neuroendocrinology, Department of Human Genetics, KU Leuven, Leuven, Belgium; Stanford University, United States of America

## Abstract

Genetic studies of rare diseases can identify genes of unknown function that strongly impact human physiology. Prolyl endopeptidase-like (PREPL) is an uncharacterized member of the prolyl peptidase family that was discovered because of its deletion in humans with hypotonia-cystinuria syndrome (HCS). HCS is characterized by a number of physiological changes including diminished growth and neonatal hypotonia or low muscle tone. HCS patients have deletions in other genes as well, making it difficult to tease apart the specific role of PREPL. Here, we develop a PREPL null (PREPL^−/−^) mouse model to address the physiological role of this enzyme. Deletion of exon 11 from the *Prepl* gene, which encodes key catalytic amino acids, leads to a loss of PREPL protein as well as lower *Prepl* mRNA levels. PREPL^−/−^ mice have a pronounced growth phenotype, being significantly shorter and lighter than their wild type (PREPL^+/+^) counterparts. A righting assay revealed that PREPL^−/−^ pups took significantly longer than PREPL^+/+^ pups to right themselves when placed on their backs. This deficit indicates that PREPL^−/−^ mice suffer from neonatal hypotonia. According to these results, PREPL regulates growth and neonatal hypotonia in mice, which supports the idea that PREPL causes diminished growth and neonatal hypotonia in humans with HCS. These animals provide a valuable asset in deciphering the underlying biochemical, cellular and physiological pathways that link PREPL to HCS, and this may eventually lead to new insights in the treatment of this disease.

## Introduction

Interest in the prolyl endopeptidase-like gene (*PREPL*) was sparked by the discovery that this gene associated with 2p21 deletion syndrome [1], [2] and also with hypotonia-cystinuria syndrome (HCS) [3]–[5]. HCS presents clinically as decreased muscle tone (hypotonia) as well as excess cystine in the urine (cystinuria), but other symptoms such as diminished growth and mild mental retardation are also associated with HCS. Invariably, HCS is associated the deletion of other genes besides *PREPL*. Often a *PREPL* neighboring gene called solute carrier family 3 (amino acid transporter heavy chain) member 1 (*SLC3A1*) is also missing. In addition, in some atypical HCS cases, *PREPL* and *SLC3A1* are deleted with another gene, calmodulin-lysine N-methyltransferase (*CAMKMT* or *C2ORF34*) [6]. Isolated deletions of *SLC3A1*, which is highly expressed in the kidney [7], are known to cause cystinuria [8], suggesting that PREPL is accountable for the other symptoms associated with HCS. More specifically, *PREPL* deletion is thought to cause neonatal hypotonia [3] and impaired growth [9], but this has yet to be confirmed by a deletion of *PREPL* alone.

PREPL is the most recently discovered member of the prolyl peptidase family. The most well-known prolyl peptidase, dipeptidyl peptidase 4 (DPP4), , regulates postprandial insulin levels through its degradation of the incretin glucagon-like peptide 1 (GLP-1) [10]. DPP4 is also a target for a new class of anti-diabetic drugs [11], attracting attention to this enzyme family and highlighting the importance of these proteins in physiology. Unlike the other prolyl peptidases, PREPL has not shown any peptide proteolysis *in vitro*, though it will cleave small molecule esters and fluorophosphonates [9], [12], [13]. Currently, PREPL is not thought to function as a peptidase; instead recent work suggests that PREPL utilizes protein-protein interactions to affect cell biology [14]–[16].

Genetic studies of diseases have provided a wealth of insight into molecular pathways underlying human physiology [17]–[20], and in some cases these insights lead to the development of novel therapeutics [21]. For instance, the investigation of hypercholesterolemia led to important insights into the regulation of cholesterol that has been valuable in the treatment of coronary heart disease [18]. Elucidating PREPL function may reveal new molecular pathways related to the regulation of muscle tone and impaired growth. Here, we develop a transgenic mouse that lacks PREPL to characterize the physiological function of PREPL. This model will allow us to conclusively test the hypothesis that PREPL is responsible for growth regulation and hypotonia *in vivo*, and will provide a valuable model that will eventually be used to elucidate the biochemical, cellular and physiological functions of PREPL.

## Results and Discussion

### Generation of Mice Lacking Prepl

**Figure 1:**
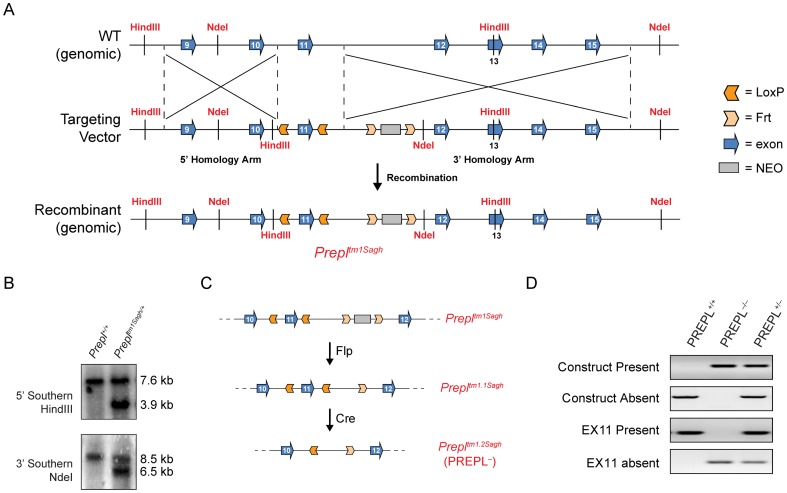
Construct design and validation. A) The genome region around PREPL's exon 11 was used to design a targeting vector for homologous recombination. This vector included a neomycin (NEO) cassette for positive selection, Frt sites flanking the NEO cassette, LoxP sites flanking exon 11, a HindIII restriction site for 5′-Southern blots, and an NdeI site for 3′-Southern blots. Homologous recombination in ES cells generated cells carrying recombinant genomic DNA, which according to standard nomenclature (*Gene^tm#Labcode^*) is referred to as *Prepl^tm1Sagh^*. These ES cells were then used to generate chimeric mice designated *Prepl^+/tm1Sagh^.* B) The germ line transmission of the *Prepl^tm1Sagh^* allele to the offspring from chimera-C57BL/6J crosses was confirmed by Southern blots of tail genomic DNA. Restriction digests of the genomic DNA with HindIII (5′) or NdeI (3′), followed by probing with a 5′ or 3′ specific probe generated a single band for *Prepl^+/+^* (left lane), whereas an additional, lower molecular weight band is generated in mice carrying the *Prepl^tm1Sagh^* allele (right lane). C) *Prepl^+/tm1Sagh^* were then crossed with mice expressing Flp recombinase to produce *Prepl^+/tm1.1Sagh^* mice (middle) lacking the NEO cassette, and finally crossed with mice ubiquitously expressing Cre recombinase, resulting in *Prepl^+/tm1.2Sagh^* mice (bottom) which lack exon 11. We refer to these *Prepl^+/tm1.2Sagh^* mice as PREPL^+/−^ mice. D) Representative PCR genotyping results for PREPL^+/+^, PREPL^−/−^ and PREPL^+/−^ mice. Using this strategy, one can easily distinguish these three genotypes as well as other possible genotypes, such as the *Prepl^tm1.1Sagh^* allele.

Generation of a mouse model began with the design and production of a targeting construct that was used to replace part of the *Prepl*-coding region of the genome in embryonic stem cells by homologous recombination. In this construct, exon 11 of the *Prepl* gene, which contains the catalytic serine nucleophile, was placed between two Cre recombinase recognition sites (LoxP sites) [22] (**Fig. 1A**). This would allow for the tissue- or age-specific removal of PREPL catalytic activity, if necessary. The construct also included additional restriction sites to enable the identification of successful homologous recombination events in ES cells and the germ line transmission of this construct in chimeric mice by Southern blot (**Fig. 1** **, Fig. S1 in File S1**). This approach circumvents any issues that might result from targeting an early exon, such as production of a truncated PREPL enzyme retaining catalytic activity. A Neomycin (NEO) selection marker was also included and flanked by flippase recognition target (Frt) sites [23], which allows removal of this cassette as well. The construct was prepared and fully sequenced to ensure accuracy and then introduced into ES cells followed by a positive selection with neomycin to identify clones that had incorporated the construct. Individual clones were then analyzed by Southern blot to identify ES cells that had undergone homologous recombination to incorporate the construct in the correct manner (**Fig. S2 in File S1**).

After karyotyping, ES cells were injected into blastocysts and implanted into mice. The resulting offspring were high percentage chimeras and germ line transmission of the new allele, *Prepl^tm1Sagh^*, was observed when breeding these animals to C57BL/6J mice (**Fig. 1A**), as determined by Southern blot analysis of the offspring (**Fig. 1A and 1B**). After excision of the Neomycin marker, these animals were crossed with mice expressing Cre recombinase to also excise exon 11 from *Prepl* (**Fig. 1C**), thus generating *Prepl^tm1.2Sagh/+^* mice, referred to from here on as PREPL^−/+^ mice. Removal of exon 11 on one allele in these offspring was confirmed by PCR. In turn, PREPL^−/+^ mice were bred to each other to produce PREPL^−/−^ and PREPL^+/+^ mice to carry out the experiments in this manuscript. A genotyping strategy was devised to distinguish between all possible genotypes in these mice, and detected the presence and absence of the construct as well as the presence and absence of exon 11 (**Fig. 1D** **, Fig. S3 in File S1**).

**Figure 2:**
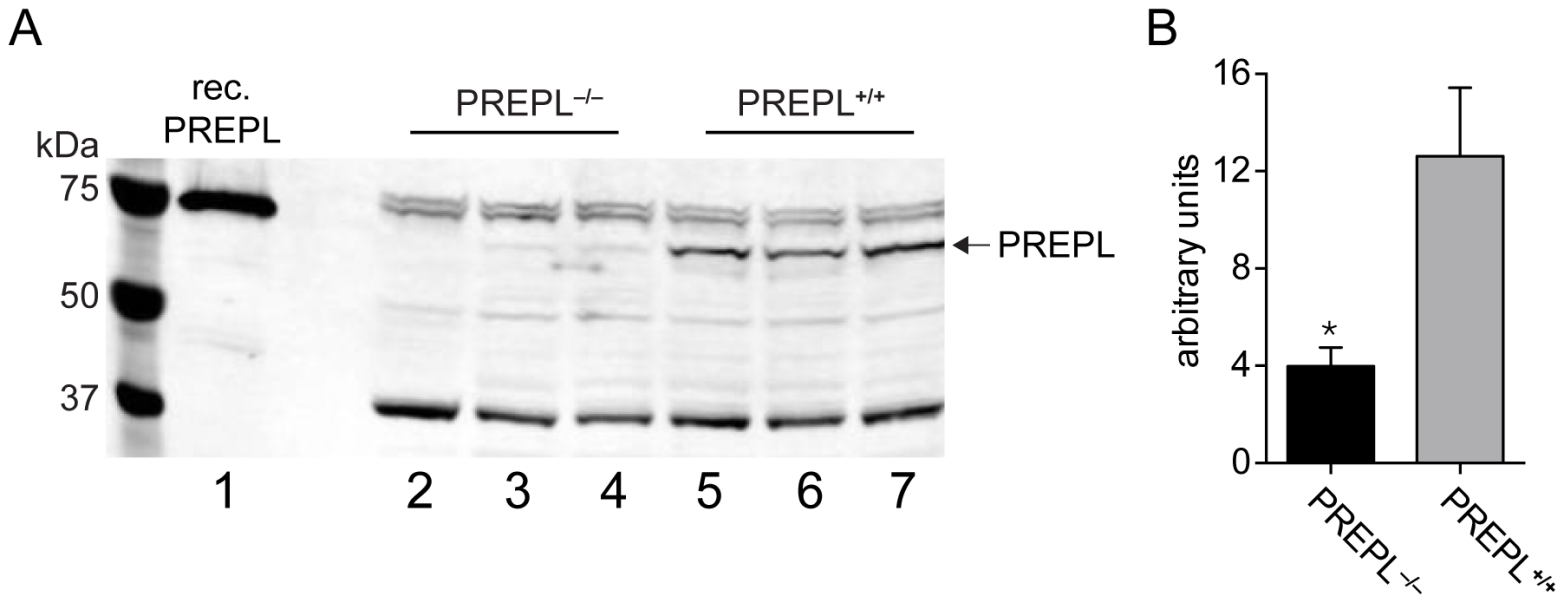
Homozygous PREPL*^−/−^* mice lack PREPL and have diminished *Prepl* mRNA levels. A) Western blot for PREPL in the spinal cords of PREPL^+/+^ and PREPL^−/−^ mice. Recombinant PREPL (lane 1) is used as a positive control to test the polyclonal antibody used to in this blot. Analysis of spinal cord samples from PREPL^−/−^ (lanes 2–4) and PREPL^+/+^ (lanes 5–7) demonstrate the PREPL^−/−^ mice lack full-length PREPL protein. Removal of exon 11 could result in a truncated PREPL protein, but no evidence of a shorter protein is evident in the western blot of PREPL^−/−^ samples (lanes 2–4). B) Analysis of *Prepl* mRNA by qPCR in mouse liver reveals that there is substantially less *Prepl* transcript in the liver of PREPL^−/−^ mice when compared to PREPL^+/+^, indicating that removal of exon 11 also impacts mRNA levels. (Error bars depict SEM. Statistical significance calculated by a Student's t-test, p-value<0.05, *, N = 4).

Next, we wanted to ensure that PREPL was in fact completely absent in this mouse. A western blot confirmed the absence of full length PREPL protein in PREPL*^−/−^* mice (**Fig. 2A**). Further, reverse-transcription-polymerase chain reaction (RT-PCR) experiments demonstrated that *Prepl* mRNA is considerably less abundant in PREPL*^−/−^* mice (**Fig. 2B**). The fact that removal of exon 11 resulted in lower levels of *Prepl* mRNA suggested that it was being degraded and analysis of the *Prepl* mRNA sequence in the absence of exon 11 showed that it will contain additional stop codons (**Fig. S4 in File S1**). To protect against the generation of truncated proteins, cells utilize a process called nonsense-mediated decay (NMD) to remove mRNAs with premature stop codons [24], and it is likely that NMD is responsible for the lower levels of *Prepl* mRNA observed in the PREPL^−/−^ mice.

### Prepl Regulates Body Size

**Figure 3:**
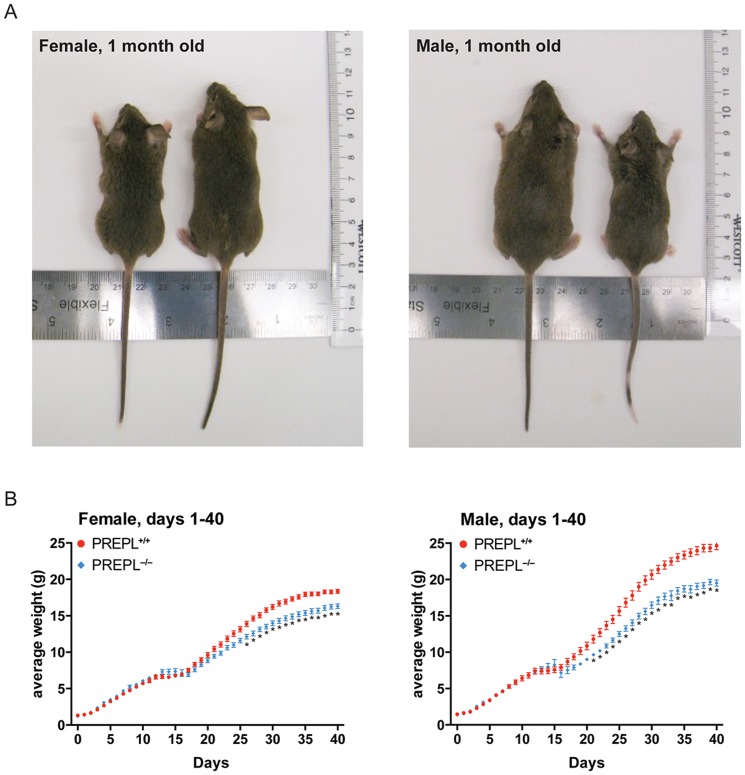
PREPL^−/−^ mice have diminished growth rates and are smaller than PREPL^+/+^ animals. A) Body lengths of one-month old PREPL^−/−^ and PREPL^+/+^ mice were compared. Both female and male PREPL^−/−^ mice are visibly smaller than PREPL^+/+^ mice. B) Female and male mice of both genotypes were weighed every day for the first forty days after birth to obtain growth curves. Female PREPL^−/−^ mice begin to gain less weight than PREPL^+/+^ mice at about three weeks old, with the data being statistically significant at day 26, while male PREPL^−/−^ mice show significantly less weight gain than PREPL^+/+^ mice from postnatal day 21. (Error bars show SEM and significance calculated using the Holm-Sidak method to correct for multiple comparisons, alpha = 5%, each row was analyzed individually, without assuming a consistent SD).

Having validated that the PREPL^−/*−*^ mice lack PREPL protein, we wanted to identify any phenotypes associated with the loss of this protein, including changes that are similar to the symptoms attributed to *PREPL* deletion in HCS. The most overt difference was that PREPL^−/−^ animals were significantly smaller than their PREPL^+/+^ counterparts (**Fig. 3**). Indeed, measurements of female and male mice at one month of age revealed that PREPL^−/−^ mice had impaired growth, with shorter body lengths compared to PREPL^+/+^ mice (**Fig. 3A**). To quantify these differences in body size, we obtained growth curves for male and female PREPL^−/−^ and PREPL^+/+^ mice from birth. PREPL^−/−^ mice of both sexes exhibited lower weights than their PREPL^+/+^ counterparts beginning around day 20 and this trend continued and increased up until the mice were 40 days old (**Fig. 3B**). Taking the area under the curve (AUC) for the respective growth curves also resulted in a significant difference for these animals between 1–40 days (**Fig. S5 in File S1**).

Humans with HCS frequently start exhibiting hyperphagia and rapid weight gain in late childhood, a phenotype suspected to be caused by *PREPL* deletion [3], [25]. To assess whether a similar phenotype was present in these animals and to determine how the weights of these mice developed over a longer time, we therefore performed more extended growth curve analysis of these animals, continuing out to 21 weeks of age (**Fig. S6 in File S1**). From this more extended growth curve, it was apparent that both male and female PREPL^−/−^ mice continued to lag behind their PREPL^+/+^ counterparts in weight later in life as well, with final weights of PREPL^−/−^ animals significantly lower than those of PREPL^+/+^ controls. This indicates that the hyperphagia observed in HCS patients is not phenocopied in the PREPL^−/−^ mice, but rather that the differences in size which first appeared at a few weeks of age remained throughout the lifetime of these animals (**Fig. S6 in File S1**).

### Neonatal Hypotonia Associated with A Loss of Prepl

**Figure 4:**
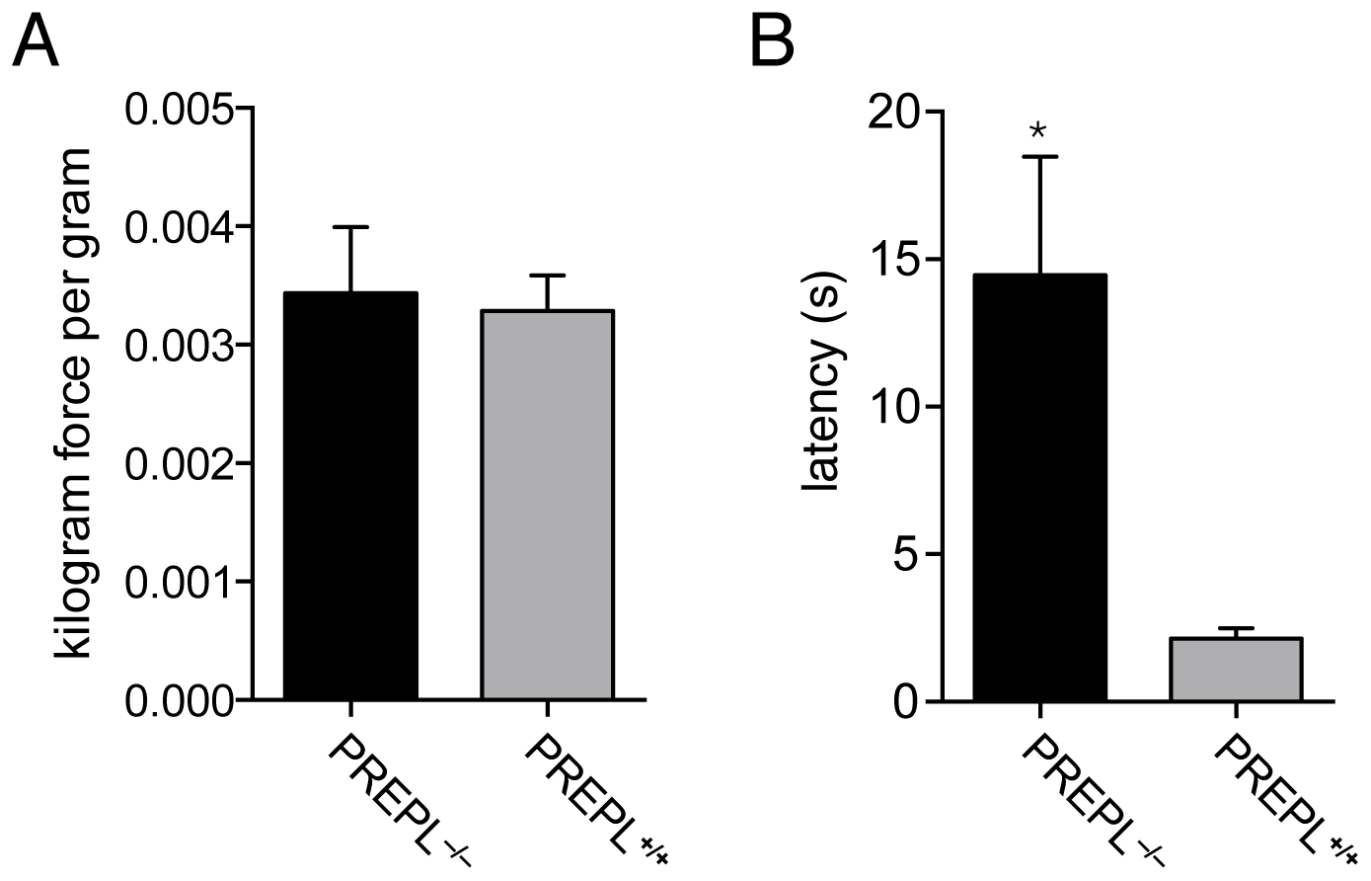
Grip strength and righting assay to assess hypotonia in PREPL^−/−^ mice. A) Six month old female mice had no difference in grip strength between genotypes when normalized to their respective body weights. B) A comparison of pups of both genders and genotypes in a righting assay. In this experiment, mice are placed on their backs and the time it takes for them to right themselves is measured. At day 5, PREPL^−/−^ pups are much slower at righting themselves than PREPL^+/+^ mice. (Error bars show SEM and Statistical significance calculated by a Student's t-test, p-value<0.05, *, N = 4–5 for grip strength and N = 13–14 for the righting assay).

Human patients with HCS typically exhibit neonatal hypotonia–decreased muscle tone [3]. In humans this is manifested by difficulties in feeding at a young age and resolves as the patients age. To determine whether neonatal hypotonia is due to PREPL deficiency, we examined these mice in two different assays. First, we performed a grip strength assay, which measures the maximum force applied by a mouse grabbing a bar with its forelimbs and is used to infer muscle strength and also hypotonia in adult mice [26], [27]. Grip strength assays on six-month old female mice found no significant difference in grip strength between PREPL^+/+^ and PREPL^−/−^ mice when normalized for body weight (**Fig. 4A**), suggesting that these animals could generate similar force per gram of mouse. Because of their smaller size, the PREPL^−/−^ mice were not as strong on an absolute scale (**Fig. S7 in File S1**).

To analyze strength and possible hypotonia at an earlier age, resembling the neonatal stage in humans, we selected a surface-righting test [28], [29] on five-day old pups. In this assay, mice are placed on their backs on a flat surface and the latency before they are able to ‘right’ themselves is measured. Hypotonic pups would be expected to take longer before being able to assume the normal upright position with all four paws on the ground than pups with normal muscle tone. Thus, this assay provides a measure of neonatal hypotonia. A surface-righting assay on PREPL^+/+^ and PREPL^−/−^ mice revealed a clear difference between these animals, with PREPL^−/−^ pups taking over twice as long to right themselves as PREPL^+/+^ mice (**Fig. 4B**). This data supports the idea that PREPL^−/−^ pups have neonatal hypotonia, and highlights a role for PREPL in the developing musculoskeletal system.

## Conclusion

Based on interest in the prolyl peptidase family and the underlying causes of HCS, we have generated a knockout model to study the physiological role of PREPL. An important goal in this study was to test the hypothesis that PREPL affects growth and muscle tone, as suggested by human genetics of individuals with HCS and 2p21 deletion syndrome [1], [2], [12]. In doing so, these experiments would also reveal whether PREPL impacts similar physiology in mice and humans, making these animals a valuable scientific model. The most overt phenotype in PREPL^−/−^ mice was their reduced body size relative to their PREPL^+/+^ siblings. Growth curves quantified this effect, demonstrating that a difference emerged at approximately three weeks and continued throughout the remainder of the animals' lives. Patients with HCS suffer from stunted growth and this mouse model demonstrates that PREPL does in fact have a role in the regulation of body size and that this function is conserved from mouse to man.

The growth curves observed for the PREPL^−/−^ mice are strikingly similar to those seen for mouse models with growth hormone defects, such as growth hormone receptor knockout mice [30], suggesting that diminished growth hormone signaling may underlie this phenotype. This theory is supported by the fact that HCS patients are known to have defects in growth hormone signaling and this has been proposed to be responsible for their stunted growth. With these PREPL^−/−^ mice in hand, it will now be possible to eventually test this hypothesis in an experimental model.

A surface-righting assay revealed that five-day old PREPL^−/−^ mice have difficulty righting themselves, indicating that these pups likely suffer from hypotonia. In humans with HCS, hypotonia is usually resolved by adulthood. While grip strength is not a direct measure of hypotonia, adult PREPL^−/−^ mice have a similar strength-to-weight ratio as PREPL^+/+^ mice, suggesting that any muscular issues have resolved by this time. In conclusion, the data from this animal model support the hypothesis that loss of PREPL is responsible for impaired growth and hypotonia in mammals, which provides additional evidence for its predicted role in HCS and 2p21 deletion syndrome. With this mouse model in hand, the molecular, cellular, and physiological mechanisms linking PREPL to growth and hypotonia can be studied in detail, which promises to reveal new pathways and mechanisms for regulating these important physiological processes.

## Materials and Methods

### Cloning of the Homologous Recombination Construct

The homologous recombination construct was cloned from a bacmid containing the C57BL/6J DNA sequence surrounding the *Prepl* gene (from the BAC library RPCI-23 from Children's Hospital Oakland Research Institute) and from the vector LoxP-Neo2 (a kind gift from Akio Kobayashi/Andy McMahon) which contains a Neomycin resistance gene under the control of a PGK promoter. The relevant portions of the genome and of the neomycin resistance marker were amplified and necessary restriction sites inserted using the following primers. **5′ Arm FWD**: AAA ACT CGA GGC GGC CGC AAA ATC ATT CAG AGC ATC CGC GTG AGG, **5′ Arm REV:** AAA AGG TAC CAC AAC CCC AAA AGC TGA TTC, **Exon 11 FWD:** AAA AGG TAC CAA GCT TAT AAC TTC GTA TAG CAT ACA TTA TAC GAA GTT ATT TGG TTT GGT TCC TAC GAT, **Exon 11 REV:** AAA AGG ATC CTT AAT TAA CAG GAT GCT GTC ACA TGG AC, **3′ Arm FWD:** AAA AGG ATC CGG CGC GCC CAT ATG CCC AGC TGC ATC TTA TTC TG, **3′ Arm REV:** AAA AGT CGA CCG GCT GGC AAG GGT GTC ATG. The 5′ Arm was cloned into the vector pSL1180DT Vector (a kind gift from Mychelle Neptune/James Horner) using KpnI and XhoI restriction sites. Correct incorporation was confirmed and the exon 11 region was cloned into this vector using BamHI and KpnI restriction sites. The 3′ Arm was the inserted using BamHI and SalI restriction sites and finally the PGK Neo cassette was incorporated using PacI and AscI restriction sites. Analytical restriction digests confirmed correct incorporation of the four sequence elements and the finished construct was completely sequenced to ensure that the sequence was correct and no mutations had been introduced (see appendix for complete sequence).

### Electroporation

An endotoxin-free maxiprep-kit (Qiagen) was used to purify sufficient amounts of endotoxin-free construct. The plasmid containing the construct was then linearized with the NotI restriction enzyme and ethanol precipitated. After washing the pellet three times with ethanol, the DNA was electroporated into the V6.5 F1 129SvJae- C57BL/6J mouse embryonic stem (ES) cell line [31], a kind gift from the Jaenisch Lab, and transformed cells were selected using standard techniques.

### Isolation of DNA for Southern blots

To prepare DNA from ES cells, 0.5 mL of PK solution (10 mM Tris (pH 8.0), 100 mM NaCl, 1 mM EDTA, 0.5% SDS, 0.2 mg/mL Proteinase K) was added to each well in a 48-well plate and incubated at 37°C for 1–4 hours. This solution was transferred to a microcentrifuge tube and extracted first with phenol:chloroform: isoamyl alcohol (49.5∶49.5∶1) and then with chloroform:isoamyl alcohol (49∶1). One milliliter of isopropanol is added to precipitate DNA, and the tubes are centrifuged for 5 minutes at 16,000 g in a microcentrifuge. The pellet is washed with 70% ethanol, which is then carefully and completely removed. The DNA is air dried for twenty minutes, whereupon it is redissolved in 100 µL of water with 0.2 mg/mL RNAse A. To prepare DNA from mouse tails, we adapted a protocol from http://www.cellmigration.org/resource/komouse/protocols/mouse_management_feb06.pdf. Briefly, 250 µL of lysis buffer (100 mM Tris (pH 8.8), 50 mM NaCl, 5 mM EDTA, 0.2% SDS, 0.2 mg/mL Proteinase K) was added to each tail and samples were incubated at 55°C overnight. Tubes were briefly centrifuged and liquid transferred to a fresh tube. Isopropanol (250 µL) was added to precipitate the DNA. The samples were inverted to mix and centrifuged at 16,000 g for 10 minutes. The supernatant was removed and the DNA was allowed to air dry for 10 minutes, after which it was suspended in water.

### Southern Blots

Ten micrograms of genomic DNA (for isolation, see section “Isolation of DNA for Southern Blots”) was digested with the appropriate restriction enzyme overnight (for 5′ Southern blots, HindIII was used, for 3′ Southern blots, NdeI was used). Digested samples were run on a 0.8% agarose TAE gel at 40 V for 3–4 hours and an image of the gel obtained. The gel was rinsed in distilled water on a rocker at room temperature for 15 minutes and depurinated by rocking in 0.25 M HCl for 30 minutes at room temperature. The dye colors should change in this process. The gel was then rinsed in distilled water on the rocker for 5–10 minutes and equilibrated in transfer buffer (0.4 M NaOH) until the dye colors to changed back to their original colors. The gel stack for transfer was set up according to the Whatman, Schleicher and Schuell Turboblotter Rapid Downward Transfer Systems manual (Whatman). The Amersham HyBond-XL membrane was used for this transfer and was presoaked in water for five minutes. The transfer was allowed to proceed overnight and an efficient transfer was assessed by determining if the blotting paper at the bottom of the stack was moist. With the gel still in place, the membrane was cut into sections along the gel wells and labeled with a pencil. The membrane was neutralized by rocking at room temperature in 20 mM Tris (pH 7), 2× SPPE for 15 minutes or more. Ten milliliters of pre-melted PreHyb solution (Clontech) was added to hybridization tubes and membranes were placed in these tubes with the DNA side pointing toward the center of tube. Membranes were rotated for 2–3 hours at 65°C in a hybridization oven while generating the probe. The 5′ probe was designed to bind 5′ of the construct, outside of the homology arm, and was generated using the following primers: **5′ Southern Probe FWD:** CCT CCA TCT CTT GGC TTT G **5′ Southern Probe REV:** GCAGT TAT TAT TCC AGG GC. The 3′ probe was designed to bind 3′ of the construct, outside of the homology arm, and was generated using the following primers: **3′ Southern Probe FWD**: GCA GTT GTT GCC ATT GTT ACT GTA GTT C and **3′ Southern Probe REV:** CCA TTT CAT GAT ACC TAG TCA TTT CAT GAT. The probes were prepared for labeling by placing 100–150 ng of the described PCR products in a final volume of 23 ul water. The DNA was denatured by boiling for 5 minutes, then cooled immediately on ice. Two microliters each of dATP, dGTP and dTTP, as well as 15 ul Random Primer Buffer Mixture (all from Invitrogen Random Primer Labeling Kit) were added to the probe. Five microliters of P32-dCTP (∼50 uCi) and 1 ul of Klenow fragment (New England Biolabs) were added and mixed by tapping, then incubated at room temperature for 2 hours. The probe was purified using a NICK column (GE Healthcare). The column was rinsed with Tris-EDTA (TE) and several columns of TE were passed through the column to equilibrate it. Sample (50 µl probe+50 µl TE, mixed) was added to the column, followed by 400 µl of TE. The eluate was discarded. Further TE (2×200 ul) was added to the column and eluates were collected. A scintillation counter was used to determine the counts per minute (cpm) for 1 ul of each eluate fraction. An appropriate volume of probe (corresponding to 35*10^6^ cpm) was removed and denatured by boiling for 5 minutes, then immediately cooled on ice. The PreHyb solution in the hybridization tubes was replaced with fresh PreHyb solution and the denatured probe added and rotated in a hybridization oven overnight. The next day, while still rotating in hybridization oven, the probe-containing PreHyb solution was discarded and the membrane washed 2×20 min with 2× SSC with 0.1% SDS, then 2×20 min with 1×SSC with 0.1% SDS (∼10 mL solution per Hyb tube). Membranes were wrapped in Saranwrap, using water as necessary to keep flat, and wrapped membranes were placed facing a phosphor storage screen in a phosphor imaging cassette. The cassette was let sit undisturbed for ∼7 days, then scanned on a phosphor imager (Storm 860, GE).

### Generation of Chimeras, Confirmation of Germ line Transmission and Removal of the Neomycin Resistance Marker and *Prepl*'s Exon 11

Blastocyst injections were carried out with two independent targeted clones to generate chimeras. High-percentage chimeras were bred to C57BL/6J mice and some of the offspring from these crosses were confirmed to harbor the construct by performing a Southern blot on DNA obtained from their tails. These *Prepl^tm1Sagh^/Prepl^+^* mice were then crossed with Pgk-Flpe mice on a Swiss Webster mixed background (a kind gift from the Andy McMahon lab) to obtain *Prepl^tm1.1Sagh^/Prepl^+^* mice. These mice, which contain the homologous recombination construct including *Prepl*'s exon 11, were then crossed with B6.FVB-Tg(Eiia-Cre)C5379Lmgd/J (stock # 003724, The Jackson Laboratory) to excise *Prepl*'s exon 11 and produce *Prepl^tm1.2Sagh^/Prepl^+^* mice (in the text referred to as Prepl+/− mice.

### PCR Genotyping

To confirm that the Neomycin marker had been excised, PCR was performed with the following primers: **NEO FWD:** CCC ACT GTC CTT TCC TAA TAA AAT GAG G, **NEO REV:** AAG ACT GTA GGG CAT TTC CGG AAC TGA. In mice were the neomycin cassette was present, this PCR produced a band, while in the absence of the neomycin resistance gene, no band was produced. Once confirmation of the removal of the neomycin resistance gene had been confirmed, genotyping was routinely performed using four different set of primers, to distinguish between all possible genotypes. The four sets were: **PRESENCE OF EXON 11 FWD:** GGTGGTGGCGAGCTAGGTCT, **PRESENCE OF EXON REV:** AGAGAGACCTATTCCCTATGTC, **ABSENCE OF EXON 11 FWD:** ACCCCTTTACCCTGTTTGTGTA, **ABSENCE OF EXON 11 REV:** AGAGAGACCTATTCCCTATGTC, **PRESENCE OF CONSTRUCT FWD:** TCTAGAAAGTATAGGAACTTCACCG, **PRESENCE OF CONSTRUCT REV:** AAGACTGTAGGGCATTTCCGGAACTGA, **ABSENCE OF CONSTRUCT FWD:** TGCCTTCAGACACCATGCTCT, **ABSENCE OF CONSTRUCT REV:** AGCTGGGCAGGATGCTG. DNA for genotyping was isolated from tail snips using the DNeasy Blood and Tissue Kit (Qiagen).

### Mouse Colony Maintenance

Animals were kept on a 12-h light, 12-hour dark schedule and fed *ad libitum*. Mice were bred either in static breeding pairs, in duos or trios. Pups were weaned at P21. Tattooing (ATS-3 General Rodent Tattoo System, AIMS) and genotyping was performed at week 5–8. All animal care and use procedures were in strict accordance with the standing committee on the use of animals in research and teaching at Harvard University and the National Institutes of Health guidelines for the humane treatment of laboratory animals. All protocols were approved by the Harvard IACUC. If during the course of these experiments mice that showed any signs of illness, including decreased activity, hunched posture, poor/decreased grooming, poor response to stimulation, and diarrhea they were euthanized in an effort to minimize their pain and distress.

### Western Blots

Spinal cords from wild type and knockout mice (as determined by PCR genotyping) were dounce homogenized in PBS containing protease inhibitors, then sonicated. Lysates were centrifuged at 20,000 g for 30 minutes at 4°C. Protein concentrations were determined by Bradford Assay and normalized. After adding loading dye, proteins were separated by SDS-PAGE, then transferred to a PVDF membrane for 90 minutes at 50 V. The membrane was blocked in Blocking Buffer (Rockland), then incubated at room temperature with PREPL primary antibody (Polyclonal PREPL antibody was generated by Open Biosystems in rabbits using a peptide epitope (EELGLDSTDAFEALKKYLKF) derived from murine PREPL) overnight at 4°C. The membrane was then washed 3× with TBS-T for 5 minutes each, followed by incubation with secondary antibody (IRDye 680LT Donkey Anti-Rabbit (LICOR)) at room temperature for one hour. After washing 3×, the western blot was imaged on a LICOR Odyssey imager.

### RT-PCR

To determine levels of *Prepl* transcripts in liver, RT-PCR was performed. For *Prepl*, the QT00143689 primer set (Qiagen), which amplifies a 113-bp amplicon covering parts of exons 6 and 7, was used. GAPDH was used as a reference gene, with the following primers: GAPDHfwd **CTC CAC TCA CGG CAA ATT CA**, GAPDHrev **ATG GGC TTC CCG TTG ATG A** [27]. Livers from *Prepl^−/−^* and *Prepl^+/+^* mice (as determined by PCR genotyping) were harvested and snap frozen in liquid nitrogen. RNA was extracted using the RNeasy kit (Qiagen), with the following adaptations: a small piece of liver was Dounce homogenized in 600 µL of the RLT buffer (10 µL β-mercaptoethanol added per mL), then passed through a 21-gauge needle 10 times on ice. After this, the protocol in the RNeasy kit was followed. RNA concentrations were determined by Nanodrop (Thermo Scientific) and normalized to 100 ng/µL. RNA integrity was assessed by agarose gel. Genomic DNA removal and reverse transcription were performed with the QuantiTect Reverse Transcription Kit (Qiagen) according to the manufacturer's instructions. PCR was carried out using the Quantitect SYBR Green PCR Kit according to the manufacturer's instructions. No template control wells were included and ROX was used a reference dye. The MX3000p thermocycler (Stratagene) was used, with the first segment comprising 15 minutes at 95°C, the second segment comprising 30 sec at 95°C, 1 min at 55°C, 1 min at 72°C for 40 cycles, and the third and final segment comprising 1 min at 95°C, 30 sec at 50°C, 30 sec at 99°C. Relative concentrations of *Prepl* mRNA was determined using the formula 100*2^(Ct(PREPL)-Ct(GAPDH)^, where C_t_(PREPL) or C_t_(GAPDH) is the average cycle threshold for 3 technical replicates with a given sample and primer set.

### Growth Curves

*Prepl^−/−^* and *Prepl^+/+^* mice were sexed and weighed every day from the day of birth until postnatal day 40. They were weaned at day 21. Beyond P40, mice were weighed every week to obtain long-term growth curves.

### Grip Strength

To measure grip strength, we used the Chatillon Grip Strength Meter to measure forelimb grip strength. The grip strength meter was set to T-PK to measure the maximum force exerted as mice were gripped by the tail, allowed to grab onto the grid with both forelimbs, then gently pulled away with a constant speed until the grasp is broken. The measurement was repeated five times for each animal, with a one minute break between measurements. The average maximum force over the five trials was calculated for each mouse and normalized to the weight of the mouse. The animals tested were 6 month old females, not backcrossed and their genotypes had been determined by PCR genotyping.

### Righting Task

Young pups (5–6 days), were placed on their backs on a clean and flat surface, and the time required for them to assume their normal upright posture with all four paws on the ground was measured.

## Supporting Information

File S1 **This file includes supporting information figures S1–S7.**
(DOCX)
