# Supplementary material for: Deletion of Prepl Causes Growth Impairment and Hypotonia in Mice
Source: PLoS One. 2014 Feb 28;9(2):e89160. doi: 10.1371/journal.pone.0089160 (PMC3938459; doi:10.1371/journal.pone.0089160)
Supplement: File S1 — This file includes supporting information figures S1–S7. (DOCX) [file pone.0089160.s001.docx]

Supporting Information

Deletion of *Prepl* causes growth impairment and hypotonia in mice

Anna Mari Lone^1^, Mathias Leidl^1^, Amanda K. McFedries^1^, James W. Horner^2,3,4^, John Creemers^5^ and Alan Saghatelian^1^

1. Department of Chemistry and Chemical Biology, Harvard University, Cambridge, Massachusetts, USA

2. Belfer Institute for Applied Cancer Science, Dana-Farber Cancer Institute, Harvard Medical School, Boston, Massachusetts, USA

3. Department of Medical Oncology, Dana-Farber Cancer Institute, Harvard Medical School, Boston, Massachusetts, USA

4. Institute for Applied Cancer Science, University of Texas MD Anderson Cancer Center, Houston, Texas, USA

5. Laboratory for Biochemical Neuroendocrinology, Department of Human Genetics, KU Leuven, Leuven, Belgium


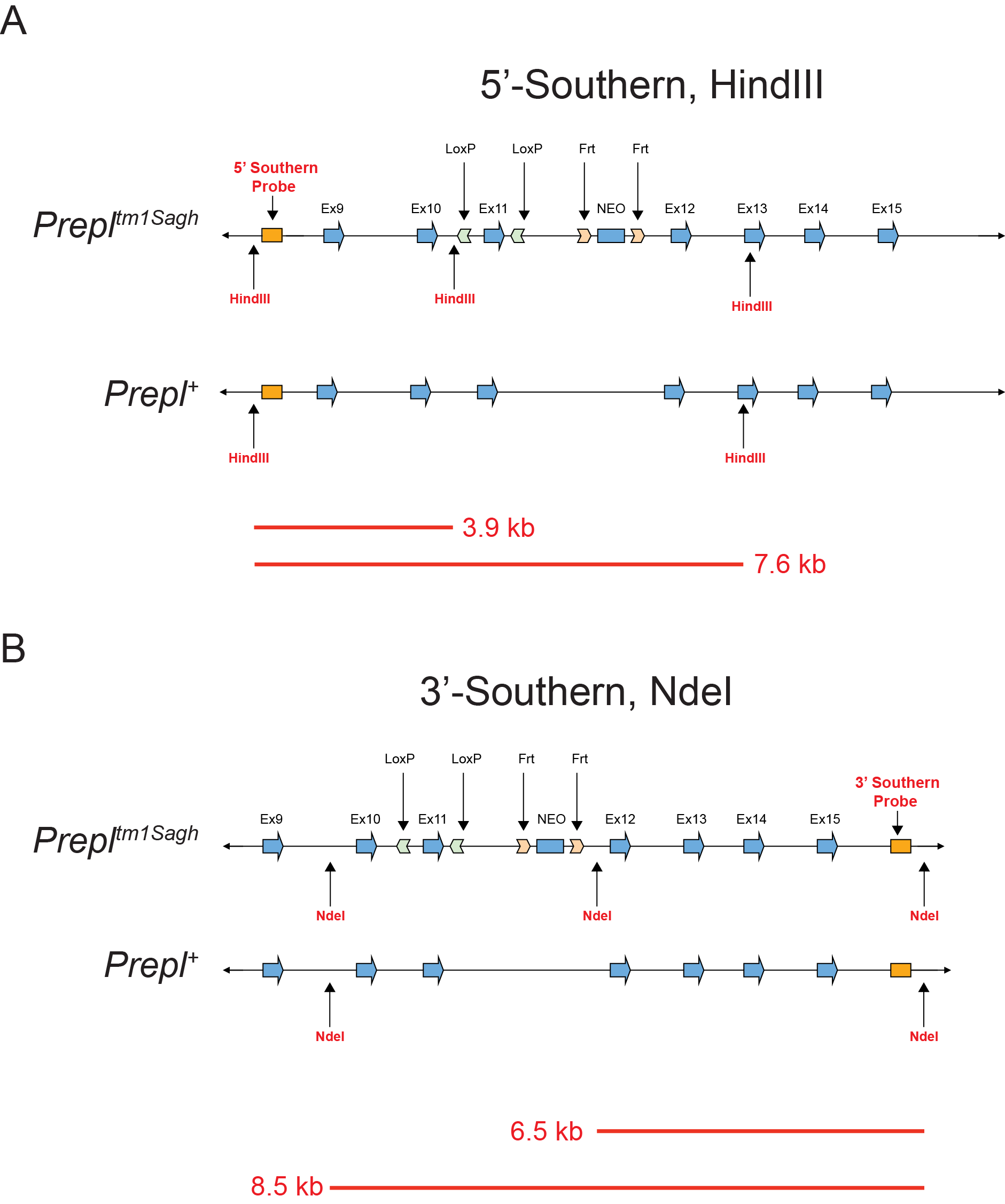


**Figure S1. 5’- and 3’-Southern strategy to ensure successful homologous recombination.** A) Homologous recombination on the 5’-side of the construct was assessed by HindIII digestion of genomic DNA. Digested DNA was analyzed by Southern blot using a radiolabeled (^32^P) 5’-Southern probe. A 7.6 kb fragment would result from the *Prepl^+^* allele whereas the *Prepl^tm1Sagh^* allele would generate a shorter, 3.9 kb, fragment due to the introduction of a new HindIII cleavage site in the targeting vector. B) A similar strategy was used for the 3’-end of the construct except that the genomic DNA was digested using the restriction enzyme NdeI. A 8.5 kb fragment results in the case of the *Prepl^+^* allele, versus a 6.5 kb fragment for the *Prepl^tm1Sagh^* allele.


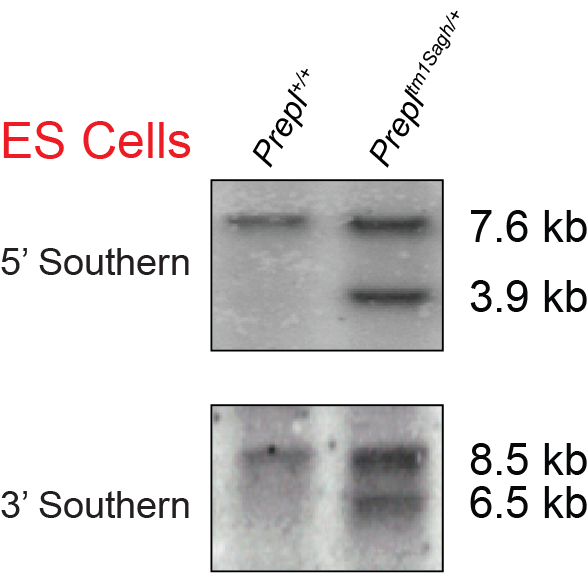


**Figure S2.** **Confirmation that ES cells contain the *Prepl* construct.** The successful targeting of ES cells with the *Prepl^tm1Sagh^* construct was confirmed by Southern blots of neomycin-resistant ES cell clones. Restriction digests of the genomic DNA with HindIII (5’) or NdeI (3’), followed by probing with a 5’ or 3’ specific probe generated a single band for *Prepl^+/+^* (left lane), whereas an additional, lower molecular weight band is generated in cells carrying one copy of the *Prepl^tm1Sagh^* allele (right lane).


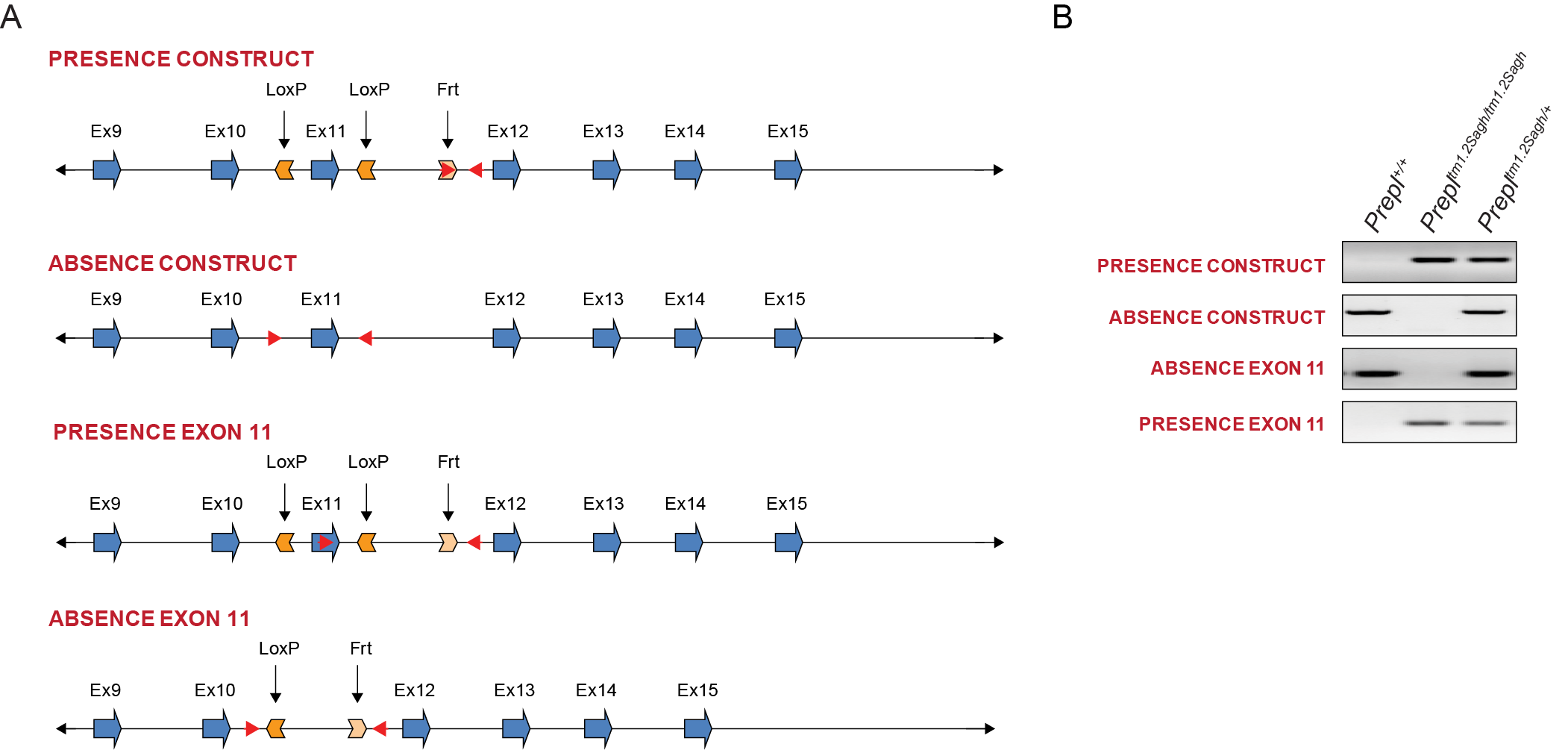


**Figure S3. PCR genotyping strategy and representative results.** A) Four different PCR reactions are used to genotype the mice. A red arrow on the genome map depicts the primers used to identify the presence/absence of construct, and the presence/absence of exon 11. The presence of construct PCR relies on a forward primer that binds to the Frt site, which is only present if the construct is integrated in the genome. The absence of construct PCR utilizes a reverse primer that spans the region of DNA where LoxP and Frt sites are inserted and is designed to only bind and amplify the wildtype allele. The presence of exon 11 PCR uses a forward primer that binds to a region within exon 11 and therefore requires exon 11 to afford a PCR product. The absence of exon 11 PCR only produces a low molecular weight band if exon 11 is absent on at least one allele. B) Representative PCR genotyping results are shown for mice not carrying the construct (PREPL^+/+^), mice carrying the construct without exon 11 on both alleles (PREPL^-/-^) and mice carrying the construct without exon 11 on one allele (PREPL^+/-^). This genotyping strategy can also be used to distinguish mice carrying one or two alleles of the construct with exon 11 intact, namely *Prepl^tm1.1Sagh/+^* and *Prepl^tm1.1Sagh/tm1.1Sagh^*.

Exon 10:

GATGGAAAGTTAGTGCCCATGACAGTTTTTCACAAAACCGATTCTGAGGACCTGCAGAGGAAGCCTCTCTTGGTGCACGTCTATGGAGCGTACGGAATGGACCTGAAAATGAATTTCAGGCCTGAAAAGCGGGTCTTGGTGGATGATGGATGGATCTTAGCTTATTGCCATGTTAG

Exon 11:

GGGTGGTGGCGAGCTAGGTCTCCAGTGGCATGCCGATGGCCGACTAACTAAAAAGCTCAATGGCCTTGCTGACTTAGTGGCTTGCATTAAGACGCTTCACAGCCAGGGCTTTTCTCAGCCGAGCCTAACAACGCTGAGCGCTTTCAGTGCTGGAGGTGTGCTCGTGGGAGCACTGTGTAATTCTAAGCCAGAGCTCCTGAGAGCCGTGACCCTGGAG

Exon 12:

GCACCTTTCTTGGATGTCCTCAATACCATGTTGGATACCACCCTTCCTCTGACACTGGAAGAGTTGGAAGAGTGGGGGAACCCGTCATCTGATGAGAAACACAAGAACTACATAAAGCGCTACTGTCCCTGCCAGAACATTAAGCCTCAG

Exon 13:

CATTATCCTTCAGTTCACATCACAGCCTATGAAAACGATGAGCGAGTGCCGCTGAAAGGAATCGTGAACTACACAGAGAAGCTTAAGGAGGCCGTGGCTGAGCACACCAAGGGAGCGGGTGAAG

Exon 14:

GCTATCAGCCCCCCAACATTATCCTAGATATTCAGCCTGGGGGGAACCACGTGATTGAGGATTCTCACAAAAAG

Exon 15:

ATCACAACCCAGATGAAATTCCTATATGAGGAACTTGGACTTGACAGCACTGATGCTTTCGAGGCGCTGAAGAAATACCTAAAGTTCTGA

Translation of exons 10-15:

D G K L V P Met T V F H K T D S E D L Q R K P L L V H V Y G A Y G Met D L K Met N F R P E K R V L V D D G W I L A Y C H V R G G G E L G L Q W H A D G R L T K K L N G L A D L V A C I K T L H S Q G F S Q P S L T T L S A F S A G G V L V G A L C N S K P E L L R A V T L E A P F L D V L N T Met L D T T L P L T L E E L E E W G N P S S D E K H K N Y I K R Y C P C Q N I K P Q H Y P S V H I T A Y E N D E R V P L K G I V N Y T E K L K E A V A E H T K G A G E G Y Q P P N I I L D I Q P G G N H V I E D S H K K I T T Q Met K F L Y E E L G L D S T D A F E A L K K Y L K F **Stop**

Translation of exons 10,12-15 (i.e. no exon 11):

D G K L V P Met T V F H K T D S E D L Q R K P L L V H V Y G A Y G Met D L K Met N F R P E K R V L V D D G W I L A Y C H V R H L S W Met S S I P C W I P P F L **Stop** H W K S W K S G G T R H L Met R N T R T T **Stop** S A T V P A R T L S L S I I L Q F T S Q P Met K T Met S E C R **Stop** K E S **Stop** T T Q R S L R R P W L S T P R E R V K A I S P P T L S **Stop** I F S L G G T T **Stop** L R I L T K R S Q P R **Stop** N S Y Met R N L D L T A L Met L S R R **Stop** R N T **Stop** S S

**Figure S4. Protein-coding sequence of the *Prepl* mRNA without exon 11.** The DNA sequence for exons 10-15 of the *Prepl* gene were translated with the EXPASY translate tool. Normal translation of exons 10-15 results in the C-terminal sequence of PREPL with a single stop codon (red) at the end of PREPL. By contrast, if only exons 10, 12, 13, 14, and 15 are translated (i.e. if exon 11 is missing) the resulting sequence is frameshifted and results in a number of additional stop codons (red). Multiple stop codon is frequently an indication of mistranslated mRNAs and these mRNAs become subject to nonsense-mediated decay, which degrades them.


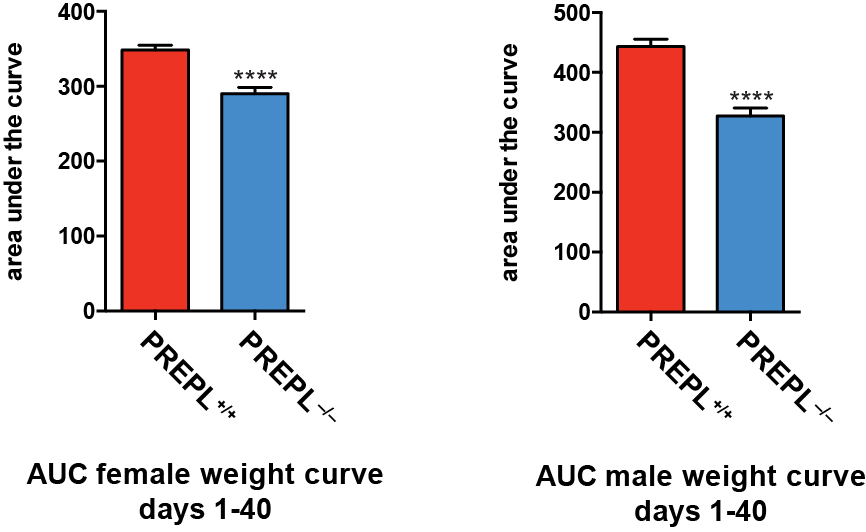


**Figure S5. Area under the curve (AUC) for weight curves (days 1-40).** Calculation of the AUCs for the weight curves between days 1-40. The data for each mouse was used to calculate an AUC and then averaged to generate these bar graphs. (Error bars show SEM; Student’s unpaired t-test; two-tailed; ****, p-value < 0.0001)


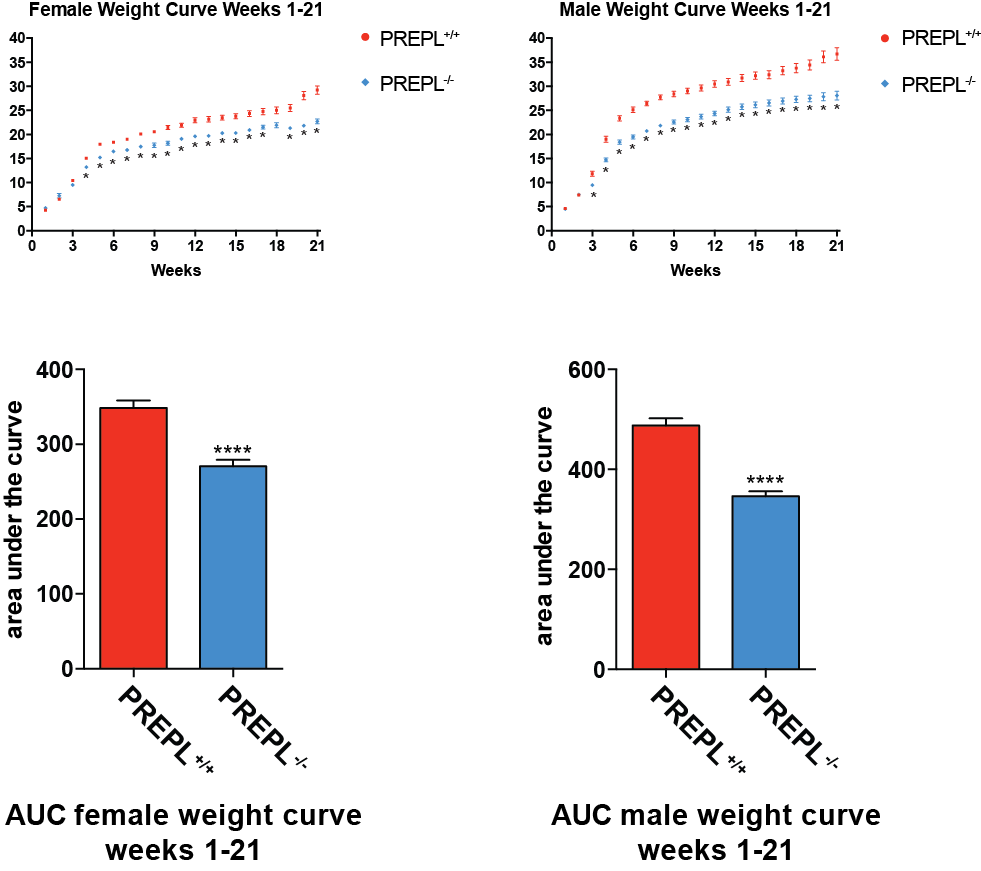


**Figure S6. Growth curves and AUCs for male and female PREPL^+/+^ and PREPL^–/–^ mice between weeks 1-21.** PREPL^–/–^ mice are smaller than their PREPL^+/+^ counterparts and this difference in size is maintained throughout their lifetimes. (For the growth curve: error bars show SEM and significance calculated using the Holm-Sidak method to correct for multiple comparisons, alpha=5%, each row was analyzed individually, without assuming a consistent SD. For the AUC: Error bars show SEM; Student’s unpaired t-test; two-tailed; ****, p-value < 0.0001).


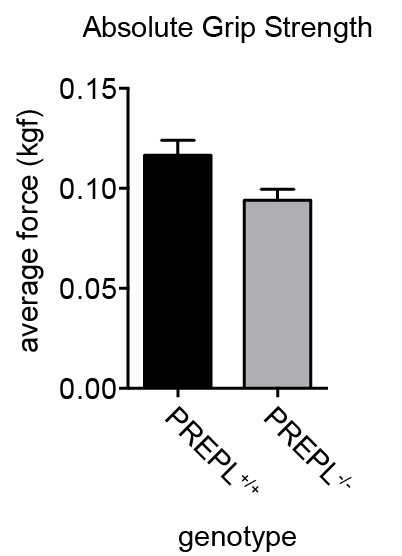


**Figure S7. Absolute values for grip strength.** On an absolute scale PREPL^–/–^ mice are weaker than their PREPL^+/+^ counterparts. (Error bars show SEM; Student’s unpaired t-test; two-tailed; p-value = 0.054)
